# Lung Volume Recruitment in Multiple Sclerosis

**DOI:** 10.1371/journal.pone.0056676

**Published:** 2013-01-31

**Authors:** Nadim Srour, Carole LeBlanc, Judy King, Douglas A. McKim

**Affiliations:** 1 The Ottawa Hospital Research Institute, Clinical Epidemiology Program, Ottawa, Ontario, Canada; 2 The Ottawa Hospital, Department of Medicine, Ottawa, Ontario, Canada; 3 The University of Ottawa, Ottawa, Ontario, Canada; 4 CANVent Program (Canadian Alternatives in Non-invasive Ventilation), The Ottawa Hospital Rehabilitation Centre; University of Pittsburgh, United States of America

## Abstract

**Introduction:**

Pulmonary function abnormalities have been described in multiple sclerosis including reductions in forced vital capacity (FVC) and cough but the time course of this impairment is unknown. Peak cough flow (PCF) is an important parameter for patients with respiratory muscle weakness and a reduced PCF has a direct impact on airway clearance and may therefore increase the risk of respiratory tract infections. Lung volume recruitment is a technique that improves PCF by inflating the lungs to their maximal insufflation capacity.

**Objectives:**

Our goals were to describe the rate of decline of pulmonary function and PCF in patients with multiple sclerosis and describe the use of lung volume recruitment in this population.

**Methods:**

We reviewed all patients with multiple sclerosis referred to a respiratory neuromuscular rehabilitation clinic from February 1999 until December 2010. Lung volume recruitment was attempted in patients with FVC <80% predicted. Regular twice daily lung volume recruitment was prescribed if it resulted in a significant improvement in the laboratory.

**Results:**

There were 79 patients included, 35 of whom were seen more than once. A baseline FVC <80% predicted was present in 82% of patients and 80% of patients had a PCF insufficient for airway clearance. There was a significant decline in FVC (122.6 mL/y, 95% CI 54.9–190.3) and PCF (192 mL/s/y, 95% 72–311) over a median follow-up time of 13.4 months. Lung volume recruitment was associated with a slower decline in FVC (p<0.0001) and PCF (p = 0.042).

**Conclusion:**

Pulmonary function and cough decline significantly over time in selected patients with multiple sclerosis and lung volume recruitment is associated with a slower rate of decline in lung function and peak cough flow. Given design limitations, additional studies are needed to assess the role of lung volume recruitment in patients with multiple sclerosis.

## Introduction

Multiple sclerosis (MS) is an inflammatory disease of the central nervous system, leading to neuron demyelination and muscle weakness. Respiratory problems in patients with MS can be due to respiratory muscle weakness, bulbar dysfunction or abnormalities of breathing control. [1] Reduced muscle strength and spasticity result in lower lung volumes and, with progression, stiffness of the lung and chest wall due to a diminished range of motion. [2] With the reduction in mobility, loose connective tissue within muscles is converted to dense connective tissue with an increase in elastance. The result is an increased work of breathing for a muscle system which is already weakened. Furthermore, loss of lung volume and resulting atelectasis diminishes alveolar surfactant, the most important element preventing alveolar collapse. Alveolar stretch is the most potent stimulus for the production of surfactant, thereby maintaining lung compliance and gas exchange. [3].

Indeed, studies have shown a reduction in several respiratory function parameters in patients with MS, including maximal inspiratory pressure (MIP), maximal expiratory pressure (MEP), forced vital capacity (FVC), forced expiratory volume in 1s (FEV1) and diffusion capacity (DLCO). [4]–[7] Respiratory function, including MIP, MEP and DLCO, was even reduced in patients with no respiratory complaints. [8] Furthermore, several studies have shown a correlation between poor respiratory function and disability in patients with MS. [4], [5], [7], [9] However, the course of respiratory function over time is not known for patients with MS.

Patients with MS are at risk for respiratory complications such as pneumonia for several reasons. First, bulbar dysfunction is associated with an increased risk of aspiration. [1] Second, the combination of reduced lung volumes, inspiratory and expiratory muscle weakness as well as glottic dysfunction results in marked impairment of cough effectiveness. [10] Compared to healthy controls, cough was found to be significantly impaired in patients with MS with lower cough volume, longer rise time and slower cough volume acceleration. [11] Peak cough flow (PCF) was also found to be inversely related to the degree of disability in a study of 27 patients with MS. [12].

PCF is an important parameter for patients with respiratory muscle weaknesss. For instance, PCF has a direct impact on airway clearance. [13] Furthermore, in a study of 49 patients with primarily neuromuscular ventilatory failure exploring predictors of successful extubation, Bach et al. have determined that a PCF of 160 L/min (2.67 L/s) was necessary for successful extubation [14] although it appeared in a further study that patients with good bulbar function could be extubated despite a PCF <160 L/min (2.67 L/s). [15] Patients with a PCF <270 L/min (4.5 L/s) may be at risk of respiratory failure following respiratory tract infections. [16]–[19].

Lung volume recruitment is a technique whereby the lungs are inflated to their maximal insufflation capacity (MIC) by the consecutive delivery, by a manual rescuscitator or volume-cycled ventilator, of volumes of air that are held with a closed glottis. [20] The MIC (FVC after lung volume recruitment) is then measured by forced exhalation through a spirometer. PCF can also be measured after a lung volume recruitment manoeuvre using a peak flow meter. Lung volume recruitment improves PCF and therefore cough effectiveness. [20] We have also found that lung volume recruitment therapy twice daily in patients with Duchenne Muscular Dystrophy dramatically improved the FVC rate of decline, a variable directly related to mortality. [21] However, the ability to increase PCF requires a sufficient difference between MIC and vital capacity, which depends on both bulbar muscle function and respiratory system compliance. Patients with either weak bulbar musculature or very low chest wall compliance may be unable to increase their VC with lung volume recruitment.

We aimed to describe the rate of decline of pulmonary function and the use of lung volume recruitment in patients with MS who presented to a specialized respiratory neuromuscular rehabilitation clinic as these are not reported in the literature.

## Methods

We collected data retrospectively from all patients with multiple sclerosis seen in the CANVent clinic (Canadian Alternatives in Non-invasive Ventilation) at the Ottawa Hospital Rehabilitation Centre from February 1999 until December 2010. Multiple sclerosis was diagnosed by the treating neurologist and patients were referred from the physiatry clinic in our institution or from other clinics for inability to manage secretions, sleep-disordered breathing, low voice volume, when they were wheelchair-assisted or for any other respiratory symptoms. This study was approved by the Ottawa Hospital Research Ethics Board, which did not require that informed consent be obtained.

The patients’ sex and age at their first visit was noted. The FVC and PCF were recorded before any manoeuvre. Lung volume recruitment manoeuvres were performed in the laboratory when patients had an FVC <80% of predicted, unless the patient refused or was unable to perform the manoeuvres due to cognitive dysfunction or physical dysfunction such as cerebellar or bulbar impairment. If lung volume recruitment manoeuvres were performed, the MIC was measured as well as the resulting PCF (PCF_LVR_). Patients were prescribed lung volume recruitment therapy at least twice daily if it resulted in improvement in pulmonary function in the laboratory. All patients used a manual resuscitation bag. None used a volume-cycled ventilator. A mouthpiece was used as the interface unless oral weakness required the use of a full face mask. The patients were instructed to squeeze the bag a sufficient number of times until they felt there lungs were full. The procedure was to be repeated for 5 full lung inflations in each session. MIP and MEP measurements were also collected. FVC and MIC values were measured with a spirometer (Profiler and CPSF/D; Medical Graphics Corporation; St. Paul, MN, US) with predicted values from NHANES III. [22] MIP and MEP were measured with a Micro RPM (Micro Medical Ltd., Bassingstoke, UK.) with predicted values by Black and Hyatt. [23] MIP was measured beginning at residual volume and MEP was measured at total lung capacity. PCFs were measured with a peak flow meter (Mini-Wright; Clement Clarke International LTD; Edinburgh Way Harlow, Essex UK).

### Statistical Analysis

SAS 9.3 statistical software (SAS Institute Inc., Cary, North Carolina) was used for all analyses. A statistical significance level of 0.05 was chosen for all analyses. Descriptive statistics were used for baseline parameters. Patients in whom a lung volume recruitment manoeuvre was performed were classified into 2 groups: a group where PCF following lung volume recruitment was higher than prior to the manoeuvre (PCF_LVR_ >PCF) and a group where there was no increase in PCF with the manoeuvre (PCF_LVR_ ≤PCF). Student’s t-test was used to compare continuous variables and Fisher’s exact test was used to compare sex between the two groups.

SAS Proc MIXED was used to build mixed-effects linear models in order to assess the rates of decline of FVC and PCF for all patients. We also built mixed-effects linear models to assess the rates of decline of FVC, PCF, MIC and PCF_LVR_, including only observations where all parameters were present in order to facilitate comparison.

Finally, mixed-linear models including baseline FVC or PCF as a covariate and group × time interaction terms were used to compare the rates of decline of FVC and PCF respectively between the 2 groups defined above. Sex and age at baseline were not included as covariates as they were not found to be significant predictors. We then added in EDSS (Expanded Disability Status Scale), disease-modifying treatment, medication for spasticity, and scoliosis, separately to these mixed-effects models to assess their association with baseline FVC and PCF. We subsequently added time interaction terms for EDSS, disease-modifying treatment, medication for spasticity, and scoliosis, separately to the mixed-effects models to assess their association with the rate of decline of FVC and PCF.

## Results

There were 79 patients included in the cohort (Table 1), 35 (44.3%) of whom were seen more than once. Among patients seen more than once, the median number of visits was 2 (IQR 2-4) with a median follow-up of 13.4 months (IQR 4.4–58.9). No patient was on invasive ventilation during the follow-up period. One patient was on non-invasive ventilation for about 5 of 10 years of follow-up. Kyphoscoliosis was present in 27/73 patients (37.0%). Patients were significantly disabled with a mean EDSS of 7.33 (95% CI 6.97–7.70). Few were on disease-modifying therapy, which included glatiramer acetate and interferon β-1a. No patient was on chronic corticosteroid therapy. Fifty patients (67.6%) were on medication for spasticity. Overall, 64 patients (82%) had a baseline FVC <80% of predicted and 63 patients (80%) had a baseline PCF <270 L/min (4.5 L/s). Measurements following lung volume recruitment were obtained in 39 patients with an FVC <80% of predicted and, at their request, in 2 patients with an FVC of 81.4% and 87.6% of predicted respectively.

**Table 1:** Patient characteristics at the first visit.

Variable	N	Value
Age (years)	79	54.9 [52.4–57.4]
Male	79	36 (45.6%)
Kyphoscoliosis	73	27 (37.0%)
EDSS	51	7.33 [6.97–7.70]
Any disease-modifying treatment	74	7 (9.5%)
Glatiramer	74	3 (4.1%)
Interferon β-1a	74	4 (5.4%)
Any spasticity medication	74	50 (67.6%)
Baclofen	74	47 (63.5%)
Dantrolene	74	10 (13.5%)
Tinzanidine	74	4 (5.4%)
Benzodiazepine	74	3 (4.1%)
FVC (L)	78	2.17 [1.93, 2.41]
FVC %	78	55.9 [50.5, 61.2]
PCF (L/s)	79	3.51 [3.07, 3.96]
MIP (cmH_2_O)	46	−39.3 [−46.9, −31.7]
MEP (cmH_2_O)	47	43.5 [35.9, 51.1]

Results are indicated as mean [95% confidence interval] or n (%) as appropriate.

Table 2 shows that the success of lung volume recruitment was not associated with age, sex, disease-modifying treatment, medication for spasticity or kyphoscoliosis. Lung volume recruitment resulted in a significant improvement in both FVC (0.62 L, p<0.0001) and PCF (0.89 L/s, p<0.0001). Patients in whom lung volume recruitment resulted in a greater PCF had a significantly lower FVC and a significantly higher MIC-VC difference. The PCF both before and after lung volume recruitment, and the MIC were not significantly different between the 2 groups.

**Table 2:** Effect of lung volume recruitment on pulmonary function at the first visit.

Variable	All (N = 41)	PCF_LVR_ >PCF (N = 30)	PCF_LVR_ ≤PCF (N = 10)	p*
Age (years)	54.3 [51.0, 57.6]	54.8 [50.6, 59.0]	52.4 [46.0, 58.8]	0.54
Sex (Male:Female)	21∶20	15∶15	5∶5	1.00
Kyphoscoliosis	19/37 (51.4%)	14/27 (51.9%)	4/9 (44.4%)	1.00
EDSS	7.55 [6.92–8.17]^a^	7.93 [7.16–8.70]^c^	7.29 [6.54–8.03]^d^	0.26
Any disease-modifying treatment	1/39 (2.6%)	1/28 (3.6%)	0/10 (0%)	1.00
Glatiramer	1/39 (2.6%)	1/28 (3.6%)	0/10 (0%)	1.00
Interferon β-1a	0/39 (0%)	0/28 (0%)	0/10 (0%)	1.00
Any spasticity medication	33/39 (84.6%)	25/28 (89.3%)	8/10 (80.0%)	0.59
Baclofen	32/39 (82.1%)	2428 (85.7%)	8/10 (80.0%)	0.64
Dantrolene	7/39 (18.0%)	5/28 (17.9%)	1/10 (20.0%)	1.00
Tinzanidine	4/39 (10.3%)	3/28 (10.7%)	1/10 (10.0%)	1.00
Benzodiazepine	2/39 (5.1%)	1/28 (3.6%)	1/10 (10.0%)	0.46
FVC (L)	1.88 [1.61, 2.14]	1.65 [1.34, 1.95]	2.43 [2.06, 2.80]	0.007
MIC (L)	2.50 [2.25, 2.75]	2.51 [2.20, 2.83]	2.59 [2.17, 3.01]	0.79
MIC-VC (L)	0.62 [0.37, 0.88]	0.87 [0.61, 1.13]	0.16 [−0.12, 0.45]	0.005
PCF (L/s)	2.82 [2.39, 3.24]^b^	2.59 [2.10, 3.08]	3.50 [2.66, 4.35]	0.058
PCF_LVR_ (L/s)	3.70 [3.20, 4.20]^b^	3.93 [3.31, 4.54]	3.04 [2.24, 3.84]	0.12
PCF_LVR_-PCF (L/s)	0.89 [0.48, 1.29]^b^	1.34 [0.93, 1.74]	−0.46 [−0.83, −0.10]	Group definition

Results are indicated as mean [95% confidence interval] or n/N (%) as appropriate.

a N = 22.

b N = 40.

c N = 14.

d N = 7.

* Comparison of patients with PCF_LVR_ >PCF with patients with PCF_LVR_ ≤PCF with Student’s t-test for continuous variables and Fisher’s exact test for categorical variables.

Among the 35 patients with longitudinal follow-up, there was a significant decline in FVC (p = 0.0007) and PCF (p = 0.003) over time at an average rate of 122.6 mL/y (95% CI 54.9–190.3) and 192 mL/s/year (95% CI 72–311) respectively.

**Figure 1:**
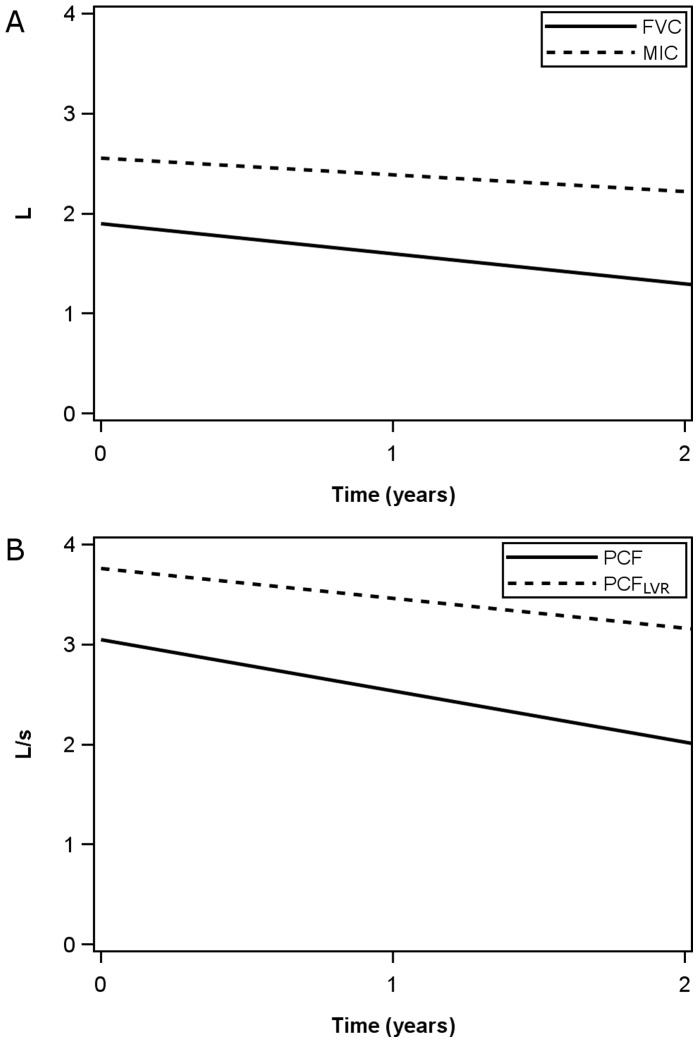
Comparative time course of average FVC, MIC, PCF and PCF_LVR_. Only observations where all variables were present were included. FVC, PCF and PCF_LVR_ declined significantly at an average rate of 89.9 mL/y, 154 mL/s/y and 89.1 mL/s/y respectively. The MIC did not decline significantly with an average rate of decline of 50.6 mL/y.

A further analysis including only observations where all variables (FVC, MIC, PCF and PCF after lung volume recruitment) were available was performed. The most common reason for exclusion was the absence of lung volume recruitment measures (49/161 observations). Four more observations were excluded and 108 observations from 53 patients were available for analysis. The FVC, PCF, and PCF after lung volume recruitment declined significantly over time (p = 0.0094, 0.0085 and <0.0001 respectively, Figure 1) but the MIC did not (p = 0.22).

The FVC rate of decline was significantly lower in those who had an improvement in PCF with lung volume recruitment at the first visit than in those without improvement (68.1 mL/y vs 299.7 mL/y, p<0.0001) as was the PCF rate of decline (110 mL/s/y vs 471 mL/s/y, p = 0.042) in an analysis where the baseline FVC or PCF respectively were included as covariates to account for differing baseline values between the groups. The EDSS (p = 0.34 and p = 0.54), the presence of disease-modifying treatment (p = 0.95 and p = 0.87), medication for spasticity (p = 0.64 and p = 0.74), and kyphoscoliosis (p = 0.37 and p = 0.58) were not significant predictors of baseline FVC or PCF. The presence of disease-modifying treatment (p = 0.99 and p = 0.91), medication for spasticity (p = 0.80 and p = 0.56) and scoliosis (p = 0.14 and p = 0.79) were not significant predictors of the rate of decline of PCF or FVC. However, the EDSS was a significant predictor of the rates of decline of FVC (p = 0.0006) and PCF (p = 0.003). Each increase in EDSS by one unit was associated with an increase in the rate of decline of FVC by 229 mL/y and PCF by 575 mL/s/y. However, in models including EDSS and EDSS × time as covariates, the rates of decline of FVC and PCF were still significantly lower in patients who had an improvement in PCF with lung volume recruitment at the first visit than in those without improvement (p<0.0001 and p = 0.0008).

## Discussion

While impairment of respiratory function has been described in multiple sclerosis, this is, to our knowledge, the first description of the decline of respiratory function over time in this population, as well as the first description of the use of lung volume recruitment in this population. We found that the FVC decreased significantly over time, more than can be expected in healthy individuals. [22] The PCF also decreased significantly over time in our selected population. Furthermore, our results suggest that individuals who demonstrate an improvement in PCF after lung volume recruitment have a lower rate of decline of both FVC and PCF compared to individuals who do not demonstrate an improvement in PCF after lung volume recruitment, despite taking into account the lower baseline values.

While the study population is selected and not representative of all MS patients, our data supports previous reports of respiratory impairment in MS patients and shows that MS patients can have substantial impairment. In fact, 82% of our patients had an FVC <80% predicted and the mean FVC was only 56% predicted. Furthermore, the PCF was also significantly reduced, with 80% of patients having a PCF below the 270 L/min (4.5 L/s) required for effective airway clearance. [16]–[19].

Similar evaluations have been made in other populations with neuromuscular diseases. Kang and Bach [20] found that half of amyotrophic lateral sclerosis patients were able to increase their lung volumes with lung volume recruitment. The remainder were unable to increase their lung volumes due to severe bulbar weakness whereas 95% of all patients with Duchenne muscular dystrophy had an MIC greater than FVC in another study. [24] By comparison, we have found that 76% of patients with MS responded to lung volume recruitment.

Since MS is a neurological disease primarily affecting lung function through muscle weakness, it is plausible that the MIC would not decline as quickly over time as the FVC. Our data is compatible with this hypothesis since the MIC in our study did not decrease significantly over time while the FVC did. Chronic underinflation does lead to decrease compliance of the lungs [2], [25] and this phenomenon could lead to some decline in MIC over time and may be prevented by regular lung inflation provided by lung volume recruitment.

Most of our patients, about three quarters, showed an improvement in their PCF with lung volume recruitment. This was associated with a concomitant increase in vital capacity. Through these effects, lung volume recruitment may facilitate airway clearance in MS patients and potentially reduce the risk of respiratory tract infection, although data about clinical outcomes was not available.

It is not surprising that those with higher baseline lung function would have less improvement with lung volume recruitment. Indeed, we found a significantly higher FVC and a trend for a higher PCF at baseline in patients who did not demonstrate improvement in PCF with lung volume recruitment. However, the rate of decline of both FVC and PCF was higher in these patients, despite taking baseline values into account. Patients who demonstrated an improvement in the laboratory following lung volume recruitment were instructed to perform the manoeuvre at least twice daily. It is therefore not possible to determine from our data whether the slower decline in lung function is due to regular performance of the manoeuvre or whether an increase in PCF following lung volume recruitment at baseline is associated with other factors affecting lung function decline.

We did try to account for other factors that could affect the rates of decline of FVC and PCF. We did not account for mechanical ventilation as there was only 1 patient on non-invasive ventilation among those followed longitudinally. We found that EDSS was a predictor of the rates of decline of FVC and PCF but disease modifying treatment, medication for spasticity or kyphoscoliosis were not. While accounting for the EDSS, the rates of decline of FVC and PCF were still significantly lower in patients who had an improvement in PCF with lung volume recruitment at the first visit than in those without improvement. It is still possible that other unmeasured baseline characteristics may account for these differences between groups.

The optimum daily frequency of lung volume recruitment is unknown. Most discussions of lung volume recruitment are directed at short term management of acute upper respiratory infection or correction of oxygen desaturation. The required frequency of lung volume recruitment for the chronic management of respiratory impairment due to muscle weakness may be different. The regimen of at least twice daily lung volume recruitment was chosen as a balance between effectiveness and practicality. The rate of decline of vital capacity was reduced significantly with twice daily lung volume recruitment in a recent study of patients with Duchenne muscular dystrophy, [21] and minimum twice daily lung volume recruitment was associated with a lower rate of decline of PCF and FVC in the present study as well.

Alternative methods to improve pulmonary function in MS have been studied. For instance, 8 weeks of expiratory muscle strength training resulted in MEP and peak expiratory flow (PEF). The PEF percentage of predicted increased from 76.5% to 104.3% (p<0.001). [11] A ten-week inspiratory muscle training program was also shown to improve MIP and various pulmonary function parameters, although cough was not assessed. [26] Given different mechanisms of action, lung volume recruitment and respiratory muscle training may be complimentary techniques to improve pulmonary function in MS.

This retrospective study does have several limitations. First, only patients referred to a respiratory neuromuscular rehabilitation clinic were studied and these patients are not representative of all patients with MS. Second, follow-up for these patients was variable, with less than half being seen more than once. Third, the effect of lung volume recruitment was not measured in all patients. Fourth, data on clinical outcomes was not available. Finally, without a control group, it was not possible to determine whether the lower rate of decline of FVC and PCF in patients who were prescribed lung volume recruitment was due to the regular performance of this manoeuvre rather than patient characteristics. For instance, intact bulbar muscle function is required for successful lung volume recruitment as the technique requires glottic control. Patients with bulbar weakness may therefore be unable to adequately perform lung volume recruitment. Differences between groups may therefore be due to this or other baseline characteristics rather than the intervention itself. Given limitations related to the study design, a prospective study of the effects of lung volume recruitment on lung function and PCF in an unselected group of consecutive MS patients is needed.
